# Do stressful life events impact long-term well-being? Annual change in well-being following different life events compared to matched controls

**DOI:** 10.3389/fpsyg.2022.1012120

**Published:** 2022-10-06

**Authors:** Chloe Howard, Nickola C. Overall, Chris G. Sibley

**Affiliations:** School of Psychology, University of Auckland, Auckland, New Zealand

**Keywords:** life events, stressors, resilience, well-being, longitudinal analysis, propensity score matching

## Abstract

Available longitudinal evidence suggests that personal growth following adversity may not be as prevalent as suggested in cross-sectional research. Firm conclusions regarding resiliency versus post-traumatic growth following adverse events are further tempered by the restricted range of outcomes assessed when examining resilience, the focus on specific adverse events or cumulative adversity scores that hinder comparisons between event types, and the relative scarcity of analyses including matched control groups. The current study addresses these gaps by leveraging longitudinal panel data comparing annual change in well-being from 2018 to 2019 for people who experienced a major life stressor relative to propensity score matched controls who did not experience such stressors over the same period. Moreover, independent comparisons are conducted across three distinct event categories: traumatic interpersonal events (*N*_*matched pairs*_ = 1,030), job loss (*N*_*matched pairs*_ = 1,361), and birth (*N*_*matched pairs*_ = 1,225), and five self-reported well-being indicators: life satisfaction, felt belongingness, self-esteem, meaning in life, and gratitude. Results indicate that people’s well-being (across all five indicators) remained consistent over the year in independent analyses of samples experiencing each of the three types of events, and did not differ from matched controls. These findings indicate high population levels of psychological resilience, in the sense that people did not decrease in annual well-being following various life events. These findings also fail to detect significant evidence for possible post-traumatic growth, insofar as such growth might relate to a broad range of different aspects of well-being.

## Introduction

The question of whether stressful events undermine well-being or lead to growth has received increasing attention. The intuitive appeal that there may be value in adversity is particularly salient given recent global turmoil, including natural disasters, mass shootings, and the COVID-19 pandemic. Despite an increased focus on potential positive change rather than just negative outcomes following trauma (see Tedeschi and Calhoun, 1996), studies assessing longitudinal changes following life events indicate that growth may not be as common as suggested in cross-sectional research (e.g., Infurna et al., 2022). Moreover, current conclusions about how resilient people are or whether they grow (in the sense of positive change) following a stressful event are limited due to the restricted range of outcomes assessed when examining resilience (Infurna and Jayawickreme, 2019), the focus on one specific adverse event or cumulative adversity scores that hinder comparisons between different types of events (Rakhshani and Furr, 2021), and the relative scarcity of analyses including matched controls to establish the causal role of life events (Mangelsdorf et al., 2019).

The current study addresses these gaps by employing longitudinal panel data comparing annual change from 2018 to 2019 for matched samples of people who experienced a major life stressor relative to propensity score matched controls who did not experience the event. To do this, we utilize two waves of annual data from the New Zealand Attitudes and Values Study (NZAVS)—a large-scale national probability panel study that provides annual assessments of important well-being indicators from large groups of people who are followed over time. The 2018 and 2019 waves also included an assessment of the natural occurrence (or not) of a variety of life events within a 1-year timeframe. We compare the well-being of people who experienced three distinct stressful event categories that vary in valence, domain, and normality—traumatic interpersonal events (*N*_*matched pairs*_ = 1,030), job loss (*N*_*matched pairs*_ = 1,361), and birth (*N*_*matched pairs*_ = 1,225)—with matched controls on five self-report well-being outcomes: life satisfaction, felt belongingness, self-esteem, meaning in life, and gratitude. Our aim is to advance understanding regarding whether stressful life events predict changes in well-being from a growth or resilience perspective.

Although there are a variety of different responses to major life events, psychological research has historically focused on negative reactions, such as by examining life events as a risk factor in the development of PTSD (Bonanno, 2004). Yet, resilience is common. The resilience literature identifies four distinct trajectories tracking change in well-being in the 2 years following a traumatic event: (1) resilience: the more common trajectory where people maintain a relatively stable level of functioning over time following adversity, (2) recovery: lower levels of functioning before returning to baseline levels, (3) delayed distress: lower levels of functioning post-event that worsen over time, and (4) chronic distress: greater distress post-event that persists over time (Bonanno, 2004; Bonanno et al., 2011).

Beyond resilience, however, recent empirical interest has shifted to post-traumatic growth (PTG; Infurna and Jayawickreme, 2019). PTG refers to the positive changes people experience from a traumatic or adverse event (Tedeschi and Calhoun, 1996) and is often measured as a multidimensional construct using the Post Traumatic Growth Inventory (PTGI; Infurna and Jayawickreme, 2019). The PTGI assess positives changes across five domains: (1) improved interpersonal relationships, (2) greater appreciation for life, (3) spiritual and/or religious growth, (4) new life possibilities, and (5) enhanced personal strength (Tedeschi and Calhoun, 1996). In contrast, resilience has been predominately assessed using one outcome, such as life satisfaction or depressive symptoms (Infurna and Luthar, 2018; Infurna and Jayawickreme, 2019), and thus conclusions about resilience assume similar resilience would occur in other unmeasured domains (Infurna and Luthar, 2018; Infurna and Jayawickreme, 2019). Yet, resilience (and perhaps growth) can vary between different domains, such as higher resilience in life satisfaction compared to other well-being (e.g., positive affect) and health outcomes (e.g., physical functioning; Infurna and Luthar, 2017). To comprehensively examine resilience or PTG following major life events requires examining a variety of different outcomes that are responsive to change.

The majority of research assessing the potential positive outcomes arising from adversity relies on cross-sectional and retrospective study designs that assess self-perceived growth following an event (Jayawickreme and Blackie, 2014; Infurna and Jayawickreme, 2019; Jayawickreme et al., 2021). Retrospective reports that use personal (and subjective) perceptions of growth pose several potential issues, such as memory and recall biases, response biases arising from PTGI items exclusively assessing positive changes after an event, which may prime participants to respond in a certain way (Jayawickreme and Blackie, 2014), and motivations of deliberative rumination and restorative justice (Harvey and Blackie, 2022). Consequently, to assess if positive or negative change occurs following a major life event, studies should capture ‘current standings’ on important well-being outcomes that are measured on different occasions over time (Jayawickreme et al., 2021). Large-scale longitudinal panel studies are particularly beneficial by following the same people over time, capturing the natural occurrence (or non-occurrence) of life events, and assessing a variety of relevant outcomes (also see Jayawickreme et al., 2021).

The importance of assessing longitudinal (positive) change following major life events is evident in studies that have tracked outcomes across time. Such studies show remarkable stability pre- and post-event across a range of outcomes, including empathy, life satisfaction, religiosity, and personality (Milojev et al., 2014; Chopik et al., 2021, 2022; Blackie and McLean, 2022; Dorfman et al., 2022; Fassbender et al., 2022; Forgeard et al., 2022; Jayawickreme et al., 2022; Reitz et al., 2022). Thus, stability or declines in functioning appear to be the more typical response to adversity, with growth or positive change being much less prevalent than suggested by prior cross-sectional research (Frazier et al., 2009; Davis et al., 2021; Rakhshani and Furr, 2021; Gander and Wagner, 2022; Infurna et al., 2022; Laceulle et al., 2022; Serrano et al., 2022). However, two primary shortcomings in current longitudinal research limit conclusions about growth or resilience. First, most studies focus on the impact of one specific adverse event or non-specific cumulative adversity scores on one specific outcome, such as personality or religiosity (e.g., Milojev et al., 2014; Davis et al., 2021; Forgeard et al., 2022). But, events vary in their characteristics and can thus impact various outcomes in different ways based on the distinct challenges and joys of the event (Infurna et al., 2022), and thus a comprehensive understanding requires assessing multiple events with differing characteristics as well as several outcomes that cover multiple well-being domains (Infurna and Jayawickreme, 2019). Second, most research lacks a matched control group who did not experience the event to determine if any change is due to the event or other factors (e.g., Davis et al., 2021; Infurna et al., 2022). Therefore, the extant research may be mistakenly attributing any observed changes in well-being over time to the event, rather than natural fluctuations or maturation processes (van Scheppingen and Leopold, 2020).

Examining the impact of specific adverse events (e.g., natural disaster; Davis et al., 2021) or cumulative adversity scores (e.g., Rakhshani and Furr, 2021) provide information about the impact of specific transitions or overall stress exposure, but overlook important differences in characteristics or types of events that require assessing multiple life events simultaneously (Rakhshani and Furr, 2021; Dorfman et al., 2022). First, event type may influence the possibility of positive changes following life events (e.g., Shakespeare-Finch and Armstrong, 2010; Karanci et al., 2012). For example, Klurfeld et al. (2020) found that those who lost a loved one reported higher spiritual change compared to those that lost their job, and El-Gabalawy et al. (2021) found that those who reported a traumatic event (e.g., sexual assault) experienced higher positive interpersonal relationships and appreciation for life compared to those who reported a negative stressor (e.g., relationship breakdown). In contrast, Fassbender et al. (2022) found that perceived valence of the event was unrelated to changes in prosociality and empathy over time, and Dorfman et al. (2022) found that wisdom remained relatively stable following different types of adversity (e.g., trauma, social conflicts, health issues).

Second, understanding growth requires not just examining longitudinal change following adverse and/or traumatic life events, but also comparing that to the change in well-being that occurs following positive experiences. For example, the Inventory of Growth after Positive Experiences (IGPE) assesses post-ecstatic growth (PEG) across four domains: (1) improved interpersonal relationships, (2) enhanced spirituality or religiosity, (3) greater meaning and purpose in life, and (4) higher self-esteem (Roepke, 2013). Preliminary evidence of positive changes following positive events has primarily focused on the event of childbirth, finding the most commonly reported positive change involved greater appreciation for life, whereas the least commonly reported involved spiritual growth (Sawyer and Ayers, 2009; Taubman-Ben-Ari et al., 2011; Sawyer et al., 2012). Examining positive events more broadly, one longitudinal study found that positive events led to more increases in optimism than negative events over the course of 7 years (Schwaba et al., 2019). Moreover, conclusions from a recent meta-analysis suggest that negative events may not have a stronger impact on PTG than positive events: positive events more strongly impacted mastery, negative events more strongly impacted interpersonal relationships, and both have equal impact on self-esteem and meaning in life (Mangelsdorf et al., 2019).

Nonetheless, the current understanding about growth following negative versus positive life events is limited because conclusions are based on *post hoc* comparisons between studies that use different samples, outcome measures, and statistical analyses (e.g., Mangelsdorf et al., 2019). These limitations primarily emerge because examining the impact of adversity is largely divorced from investigating possible positive experiences, and the exclusion of positive events in current examinations of growth precludes oft-made implications that growth is only possible following adverse experiences (Roepke, 2013). Testing whether adversity is required for growth or resilience to occur requires assessing change in several key well-being outcomes following various positive and negative events (Roepke, 2013). If growth (or lack thereof) occurs for both positive and negative life events, valence is unlikely to be an important component in growth or resilience following a stressor. Moreover, if growth is more likely to follow adversity than positive events, an additional important question is whether impact depends on the severity of the adversity.

Expanding prior research to more systematically test the idea that growth follows adversity, in the current study we examine three distinct event categories that not only differ in valence, but also in life domain and normality: traumatic interpersonal events, job loss, and birth. We selected these events to assess different levels of adversity that reflect prior PTG research (e.g., trauma versus negative stressor) as well as integrate research on positive experiences by including the positive event type of birth. To more comprehensively understand how these different types of events influence well-being, we assess their impact on five key well-being outcomes covered in both the PTG and PEG literature: life satisfaction, felt belongingness, self-esteem, meaning in life, and gratitude.

The current research also overcomes another critical methodological limitation. Many of the conclusions made about the impact of life events on well-being over time are based solely on people who experienced the event (Mangelsdorf et al., 2019; van Scheppingen and Leopold, 2020; Forgeard et al., 2022), which means that changes in well-being that naturally occur may be mistakenly attributed to the event (van Scheppingen and Leopold, 2020). Accordingly, matched control groups who did not experience the targeted event but are demographically similar to those that experienced the targeted event are required to assess whether any observed changes occurred due to experiencing an event rather than other factors or normal maturation processes (Mangelsdorf et al., 2019). Although matched controls are largely absent from the growth and resilience literature, the broader literature has recently began implementing matched controls to assess the casual role of life events. Challenging the notion that any changes are caused solely by the event, the few studies that do include matched controls showed important similarities between the event and control groups (e.g., Costanzo et al., 2009; van Scheppingen et al., 2016; van Scheppingen and Leopold, 2020). For example, one of the few studies that directly examine growth using a demographically matched (age, gender, ethnicity) control group found that although cancer survivors experienced growth across several domains (e.g., spirituality), the matched control group exhibited the same improvements in well-being (Costanzo et al., 2009).

The few existing studies that implement matched controls also have limitations by predominantly focusing on the impact of relationship transitions (e.g., divorce, parenthood) on one specific aspect of well-being (e.g., life satisfaction, self-esteem) or personality (e.g., van Scheppingen et al., 2016; van Scheppingen and Leopold, 2020). Furthermore, despite these few studies accounting for various demographic and personality correlates that relate to the occurrence of an event and well-being, there are no studies to our knowledge that account for the presence of other events that may also occur in the group who experienced the focal event but also in the control group (Mangelsdorf et al., 2019). The occurrence of other stressors beyond the focal event may bias responses among controls and limit the ability to make casual inferences about the role of experiencing an event on personal growth (Mangelsdorf et al., 2019). In the current study, we conduct a more precise test of the causal role of life events in well-being by matching participants on demographics, personality, and the presence of other stressors (including the other two focal events), and conducting comparisons across multiple events and outcomes.

In sum, important limitations of the existing literature hinder firm conclusions about resiliency or growth following stressful events, including the restricted range of outcomes assessed when examining resilience (Infurna and Jayawickreme, 2019), the focus on one specific adverse event or cumulative adversity scores that hinder comparisons between different types of events (Rakhshani and Furr, 2021), and the need to incorporate matched controls to establish the causal role of life events (Mangelsdorf et al., 2019). We overcome these limitations by comparing matched samples of people who experienced a major life stressor relative to propensity score matched controls who did not experience the same event across three distinct event categories: traumatic interpersonal events, job loss, and birth. We chose three event categories that vary in valence (e.g., birth as a more positive event and job loss as a more negative event), life domain (e.g., family versus work), and normality (e.g., trauma as rarer and job loss as more common). We compare change in well-being using a range of important well-being outcomes that cover the broad array of well-being domains, align with other studies assessing positive changes following events, and that are sensitive to change over time: life satisfaction, felt belongingness, self-esteem, meaning in life, and gratitude.

These methodological strengths are accomplished by utilizing annual data from two waves (2018–2019) of the NZAVS—a large-scale national probability panel study that began in 2009. With roughly 60,000 participants, the NZAVS measures a range of outcomes each year, including well-being, health, political and social attitudes, and personality. The 2018–2019 waves of the NZAVS are the first to incorporate the Broad Inventory of Specific Life Events (BISLE; Howard et al., 2022), which simultaneously assesses an array of specific life events occurring in the past year that are then grouped into broader categories and general life event domains. The BISLE captures the natural occurrence of both positive and negative, as well as rare and common, life events. Consequently, the NZAVS and BISLE provide a unique opportunity to assess annual differences in five well-being outcomes and three distinct event categories relative to matched controls who did not experience the event.

We focused on three distinct event categories of traumatic interpersonal events, job loss, and birth (see Table 1). We chose the three events based on (a) high and comparable frequencies in our dataset and (b) the types of events typically examined in the literature (e.g., childbirth is the most frequently examined positive event; e.g., Sawyer et al., 2012). We chose job loss to represent a more normative yet negative event given the salience of this event in our sample (i.e., high frequency count) and that job loss does not classify as trauma under the DSM-5 (i.e., direct or indirect exposure to actual/threatened death, sexual violence, or serious injury; American Psychiatric Association, 2013). Moreover, prior scales assessing growth (PTGI or IGPE) measure perceived change across several domains (Infurna and Jayawickreme, 2019), whereas little is known about resilience across multiple outcomes (Infurna and Jayawickreme, 2019). Therefore, we also examine five key well-being outcomes that are (a) measured across both waves, (b) map onto the PTGI and IGPE domains, and (c) are sensitive to change following an event (Frazier et al., 2009; Infurna et al., 2022). The five constructs we assess are (1) life satisfaction—people’s cognitive judgment on their quality of life (Diener et al., 1985), (2) gratitude—people’s responsiveness to and recognition of grateful emotions (McCullough et al., 2002), (3) felt belongingness—people’s feeling of being valued and accepted by others (Hagerty and Patusky, 1995), (4) self-esteem—people’s subjective evaluation of their personal worth (Orth and Robins, 2014), and (5) meaning in life—people’s feeling of coherence and purpose in life (Steger et al., 2006).

**TABLE 1:** Specific events included in the three event categories examined as measured using the BISLE and definitions of key constructs.

Event category	Traumatic interpersonal events	Job loss	Birth
Specific events	• Someone assaulted you, abused you, or attacked you • Someone sexually harassed you • Someone sexually assaulted you • Domestic violence • Family member attacked or assaulted • Family member experienced abuse • Bullied, stalked, or threatened (includes online) • Other traumatic interpersonal event	• Lost your job or had the principal earner in your household lose their job • Fired at work • Redundancy • Partner made redundant • Resigned from job • Partner resigned from their job • Partner lost job (principal earner not defined) • Family member lost/quit job (not principal earner)	• The birth of a child • Birth of first child • Birth of a grandchild • Birth of first grandchild • Close family member gave birth • Close friend gave birth
Definitions			
*Post-traumatic growth*	The positive changes people experience from a traumatic or adverse event (Tedeschi and Calhoun, 1996)		
*Resilience*	Maintenance of a relatively stable level of functioning over time after experiencing adversity (Bonanno, 2004)		
*Traumatic event*	Direct or indirect exposure to actual or threatened death, sexual violence, or serious injury as classified under the DSM-5 (American Psychiatric Association, 2013)		

Across outcome measures and stressors, we compare propensity-matched samples of people who reported the event with people who did not report the event (e.g., comparing those who reported a birth with those that did not report a birth). Propensity score matching allows for causal inferences (Foster, 2010; Stuart, 2010; Austin, 2011) to the extent that it allows for the approximation of a matched control group (Rosenbaum and Rubin, 1983; Thoemmes and Kim, 2011). Specifically, the three event groups were compared with equally sized control groups matched on a range of key demographic variables (e.g., gender, age, relationship status) and personality traits. We match on personality given that extraversion and neuroticism may (a) predict the occurrence of specific events (Denissen et al., 2019), (b) influence or explain how people respond to an event (Sarubin et al., 2015), and (c) predict different well-being outcomes (e.g., self-esteem; Robins et al., 2001). Participants were also matched on whether they experienced zero or one or more other prevalent event categories (including the two other events of interest) concurrent to the focal event, allowing more confidence that any observed differences in the event versus matched control groups is caused by experiencing the focal event rather than the presence of other factors or events.

## Materials and methods

### Participants and sampling procedure

The NZAVS is a national probability panel study that began in 2009 and examines a range of variables including social attitudes, life events, personality, and health and well-being outcomes each year. Participants are sampled from the electoral roll and closely represent the general New Zealand population. However, there are a few minor deviations, including that women and Europeans are overrepresented by roughly 10% and Asian people are underrepresented by roughly 5%. Data for the current study are drawn from Time 10 (2018; *N* = 47,951) and Time 11 (2019; *N* = 42,684) of the NZAVS, which are the first two waves that incorporate the BISLE and include the largest NZAVS sample sizes. Retention rates between Time 10 and Time 11 was relatively high at 72.5% for Time 11, with 34,782 participants retained from Time 10. To ensure optimal matching, participants from the total samples with missing demographic data at Time 10 and/or missing averages across the outcomes for one or both waves were excluded. To isolate the effect of experiencing an event at the first timepoint, we also excluded any participants in the control and event groups from Time 10 that reported the focal event at Time 11. Therefore, one-to-one matching occurred for 1,030 participants who reported a traumatic interpersonal event and 29,148 potential controls, 1,361 participants who reported a job loss event and 28,643 potential controls, and 1,226 participants who reported a birth event and 29,115 potential controls.

### Propensity score matching

As it is unethical to subject people to experiencing certain life events, we employ propensity score matching to disentangle the causal role of experiencing an event from other factors that may be driving any observed effect (e.g., maturation processes; Thoemmes and Kim, 2011). Using the one-to-one propensity score matching function in SPSS 27 (see Thoemmes, 2012), we matched participants who experienced an event for each of our three event categories (i.e., traumatic interpersonal events, job loss, and birth) with people who had a similar propensity score but did not report the corresponding event in the same timeframe (see Sibley et al., 2020; Cross et al., 2021). Our matching process prioritized exact matches and used a match tolerance of 0.01, which means the distance in propensity scores for two participants to be matched was restricted to 0.01 SDs of the logit of the propensity score (Austin, 2011).

For each event category, we matched participants on a range of demographics from the first wave that were associated with the occurrence of the life events and/or well-being outcomes. Specifically, participants were matched on ethnicity, gender, age, education level, having a partner, the HEXACO personality traits, and whether they experienced zero versus one or more of other prevalent life event categories (e.g., marriage, retirement, illness, accident, relationship breakdown, etc.), including the two other events of interest, at the same time point (2018) as the event in focus. Including the two other focal events in our matching process means we could isolate the effect of each type of event, as some participants may experience more than one of the events of interest (e.g., birth and job loss). In matching our participants, we aimed to produce closer matches whilst also maintaining large sample size across groups. The matching process resulted in 1,030 matches for traumatic interpersonal events (0 failures to match), 1,361 matches for job loss (0 failures to match) and 1,225 matches for birth (1 failure to match). As the matching was repeated for each event, some participants are repeated across events. Specifically, 67 participants (2.59%) are included in the event groups for both job loss and birth, 33 participants (1.46%) are repeated in the event groups for birth and traumatic interpersonal events, and 91 participants (3.81%) are repeated as reporting both a job loss and traumatic interpersonal event. Nine Participants (0.25%) were included in the event groups for all three event categories. Table 2 shows the descriptive statistics for the matching variables across the event and control groups for each event category.

**TABLE 2:** Descriptive statistics for the propensity score matching variables across event and control groups for traumatic interpersonal events, job loss, and birth.

	Traumatic interpersonal events		Job loss		Birth	
Variable	Event group (*n* = 1030)	Control group (*n* = 1030)	Event group (*n* = 1361)	Control group (*n* = 1361)	Event group (*n* = 1225)	Control group (*n* = 1225)
Age, *M (SD)*	46.73 (14.47)	46.76 (14.34)	48.42 (12.20)	48.21 (14.35)	44.98 (14.07)	44.28 (13.93)
Education (0–10), *M (SD)*	5.22 (2.68)	5.29 (2.71)	5.41 (2.72)	5.42 (2.75)	5.70 (2.57)	5.83 (2.57)
Gender (yes)						
*Women*	69.32% (714)	68.25% (703)	68.55% (933)	68.92% (938)	68.90% (844)	68.74% (842)
*Men*	30.68% (316)	31.75% (327)	31.45% (428)	31.08% (423)	31.10% (381)	31.27% (383)
Ethnicity (yes)						
*Māori*	14.85% (153)	15.05% (155)	9.99% (136)	9.18% (125)	10.45% (128)	10.69% (131)
*Pacific*	2.14% (22)	1.94% (20)	2.20% (30)	2.57% (35)	2.94% (36)	2.53% (31)
*Asian*	3.98% (41)	4.08% (42)	3.67% (50)	4.26% (58)	4.25% (52)	3.51% (43)
Partner (yes)	61.36% (632)	62.82% (647)	71.64% (975)	71.93% (979)	89.80% (1100)	87.84% (1076)
Other event categories						
*Zero*	24.08% (248)	24.66% (254)	34.17% (465)	35.05% (477)	48.74% (597)	48.65% (596)
*One or more*	75.92% (782)	75.34% (776)	65.83% (896)	64.95% (884)	51.27% (628)	51.35% (629)
HEXACO personality						
*Neuroticism*, *M (SD)*	3.84 (1.23)	3.84 (1.20)	3.67 (1.17)	3.65 (1.19)	3.46 (1.15)	3.46 (1.13)
*Extraversion*, *M (SD)*	3.99 (1.25)	4.00 (1.28)	3.96 (1.22)	3.94 (1.20)	3.98 (1.16)	3.92 (1.21)
*Openness to experience*, *M (SD)*	5.23 (1.12)	5.21 (1.08)	5.11 (1.13)	5.08 (1.09)	4.97 (1.10)	4.97 (1.11)
*Agreeableness*, *M (SD)*	5.46 (1.03)	5.45 (0.96)	5.44 (0.99)	5.45 (0.97)	5.43 (0.96)	5.44 (0.97)
*Conscientiousness*, *M (SD)*	5.04 (1.11)	5.07 (1.05)	5.06 (1.05)	5.11 (1.07)	5.15 (1.02)	5.15 (1.09)
*Honesty-humility*, *M (SD)*	5.26 (1.27)	5.25 (1.22)	5.34 (1.16)	5.34 (1.25)	5.49 (1.13)	5.55 (1.10)

### Measures

#### Life events

The BISLE (Howard et al., 2022) measures life events using a checklist of 15 common life events (e.g., lost your job, birth of a child) followed by an open-ended question asking “Finally, have you experienced any significant life events in the past year?”. Open-ended responses are coded using a simple yes/no scheme according to a coding schedule of 590 specific life events, which are then merged with the checklist events and collapsed into 141 broad event categories (e.g., job loss, birth) and then again into 22 general life domains (e.g., work, family additions). Our analyses focus on the three event categories of traumatic interpersonal events (includes sexual harassment, physical assault, etc.), job loss (includes involuntary job loss, resignation, etc.), and birth (includes birth of child, grandchild, etc.; see Table 1 for more detail). A ‘yes’ response indicates that participants reported experiencing at least one specific event within the category. Other commonly examined events that are also covered in the BISLE, such as a family bereavement, were omitted from our study because of their ambiguous categorization (i.e., could be traumatic if it was due to suicide/accident or a negative stressor if due to old age/illness) or small sample frequencies (e.g., relationship breakdown).

#### Well-being outcomes

Items for all outcomes were rated on a 1 (*strongly disagree*) to 7 (*strongly agree*) scale, except for self-esteem which was rated on a 1 (*very inaccurate*) to 7 (*very accurate*) scale. *Life satisfaction* was measured using the average of two items from the Satisfaction with Life Scale (Diener et al., 1985): “I am satisfied with my life” and “In most ways my life is close to ideal” (T10: α = 0.77, T11: α = 0.78, *r*s = 0.64, *p*s < 0.001). *Felt belongingness* was measured using the average of three items adapted from the Sense of Belonging Instrument (Hagerty and Patusky, 1995): “Know that people in my life accept and value me,” “Feel like an outsider” (reverse-coded), and “Know that people around me share my attitudes and beliefs” (T10 and T11: α = 0.60). *Self-esteem* was assessed using the average of three items from the Self Esteem Scale (Rosenberg, 1965): “On the whole am satisfied with myself,” “Am inclined to feel that I am a failure” (reverse-coded), and “Take a positive attitude toward myself” (T10: α = 0.81, T11: α = 0.82). *Meaning in life* was measured using the average of two items from the Meaning in Life Questionnaire (Steger et al., 2006): “My life has a clear sense of purpose” and “I have a good sense of what makes my life meaningful” (T10: α = 0.73, *r* = 0.58, *p* < 0.001; T11: α = 0.75, *r* = 0.60, *p* < 0.001). Lastly, *gratitude* was measured using the average of three items from the Gratitude Questionnaire (McCullough et al., 2002): “I have much in my life to be thankful for,” “When I look at the world, I don’t see much to be grateful for” (reverse-coded), and “I am grateful to a wide variety of people” (T10: α = 0.53, T11: α = 0.55).

#### Propensity score matching variables

The following variables were assessed by responses to open-ended questions coded as follows: (1) gender, “What is your gender?” (0 = women, 1 = men; Fraser et al., 2019), (2) partner, “What is your relationship status?” (0 = no partner, 1 = partner), (3) ethnicity (Māori, Pacific, and Asian), tick boxes followed by “Which ethnic group(s) do you belong to?” (Statistics New Zealand, 2020), and (4) education, “What is your highest level of qualification?” (0 = low through to 10 = high; New Zealand Qualifications Authority, 2012). Age was calculated from participants year of birth (“What is your date of birth?”). The HEXACO personality traits were measured using the 24-Mini-IPIP (Sibley, 2012). The average of four items were used to assess each trait: Neuroticism (α = 0.73), Extraversion (α = 0.76), Openness to Experience (α = 0.71), Agreeableness (α = 0.72), Conscientiousness (α = 0.69), and Honesty-Humility (α = 0.75). For other prevalent life events, we created a sum score of experiencing 12 other prevalent event categories in the BISLE: family member death, began relationship, relationship breakdown, employment changes, retirement, illness, accident/injury, marriage, moved house, loss of possessions, as well as the other two focal events (i.e., job loss and birth were included when matching traumatic interpersonal events). The sum score was then recoded to create a yes/no variable that indicates if the participant reported other prevalent event categories, including the two other events of interest, in the same timeframe as the focal event (yes, 1) or not (no, 0).

### Analytic strategy

We compared annual differences in well-being across five outcomes: life satisfaction, felt belongingness, self-esteem, meaning in life, and gratitude, for people who experienced a major life stressor (event group) and propensity score matched controls who did not experience the event (control group) across three distinct event categories: traumatic interpersonal events, job loss, and birth. By employing longitudinal panel data in 2018 and 2019, we could conduct these comparisons of well-being in the year the event was reported (2018, timepoint when participants reported whether an event occurred during the prior year) as well as post-event/adaptation assessments the following year (2019) to investigate immediate and longer-term changes in well-being. We conducted a series of 2 (event: experienced the event, matched control) × 2 (year: 2018, 2019) ANOVAs for each of the five outcomes and complete these analyses separately for each event category of traumatic interpersonal events, job loss, and birth. We cannot statistically test differences between the three event categories given that some participants are repeated across event types (for more detail, see propensity score matching procedure). Therefore, we focus our comparisons on those who did experience the event versus those who did not experience the event and explore if similar differences between groups emerge across different classes of life events. To account for multiple testing bias, we set our criteria for statistical significance to *p* < 0.01.

A significant two-way interaction between event and year would suggest that the difference between the two assessment points is different across those who did experience the event versus those who did not experience the same event. To examine this further, we conduct simple effects tests on significant event × year interactions, with Bonferroni adjustment for multiple comparisons, focusing on the multivariate tests that examine the effect of year across the two groups (i.e., is there a significant difference between the two timepoints for each group?). For growth to be demonstrated, the event group should show an increase in well-being between 2018 and 2019 that does not occur for the matched controls. If the interaction effect is not significant, this would suggest that the annual differences from 2018 to 2019 do not differ between the two groups and indicate general resilience to the event. In this case, any significant main effects across years would reflect more societal or age-related changes rather than the effect of experiencing an event. Any main effects of events would suggest that, on average, experiencing an event within the prior year was associated with higher or lower levels of well-being relative to matched controls who did not experience the event.

## Results

### Life satisfaction

**FIGURE 1:**
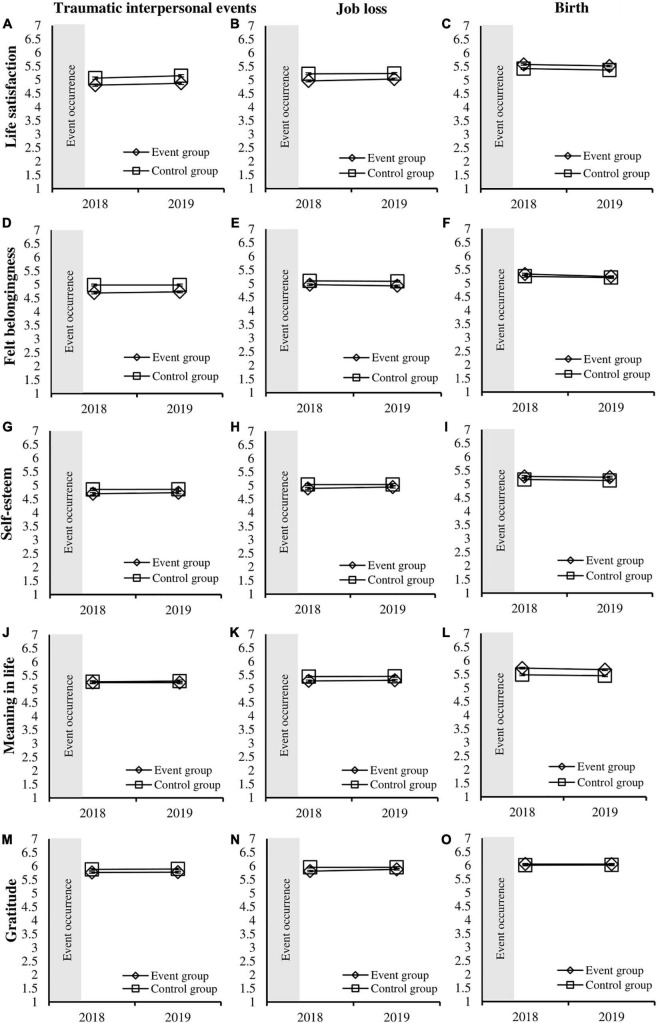
Average levels (with standard errors) of life satisfaction, felt belongingness, self-esteem, meaning in life, and gratitude in 2018 and 2019 across matched event and control groups for traumatic interpersonal events, job loss, and birth **(A–O)**.

We examined if event (experienced the event vs. matched control) and year (2018 vs. 2019) interacted to predict life satisfaction for each of three types of events: traumatic interpersonal events, job loss, and birth (see Figure 1 and Table 3). The main effects of event were significant for traumatic interpersonal events [*F*_(1,2058)_ = 23.12, *p* < 0.001, η_p_^2^ = 0.011], job loss [*F*_(1,2720)_ = 26.08, *p* < 0.001, η_p_^2^ = 0.009], and birth [*F*_(1,2448)_ = 14.20, *p* < 0.001, η_p_^2^ = 0.006], with the event groups having lower life satisfaction than the control group, except for birth where the event group was higher in life satisfaction. Moreover, main effects of year were significant for the models examining traumatic interpersonal events [*F*_(1,2058)_ = 11.35, *p* < 0.001, η_p_^2^ = 0.005] and birth [*F*_(1,2448)_ = 9.81, *p* = 0.002, η_p_^2^ = 0.004], but not significant for the model examining job loss [*F*_(1,2720)_ = 5.13, *p* = 0.024, η_p_^2^ = 0.002]. For the traumatic interpersonal events model, relative to 2018, participants showed greater life satisfaction in 2019, whereas the birth model showed that participants’ life satisfaction decreased from 2018 to 2019. However, the event × year two-way interaction was not significant for all three event types: traumatic interpersonal events [*F*_(1,2058)_ = 0.21, *p* = 0.646, η_p_^2^ = 0.000], job loss [*F*_(1,2720)_ = 2.71, *p* = 0.100, η_p_^2^ = 0.001], and birth [*F*_(1,2448)_ = 0.01, *p* = 0.926, η_p_^2^ = 0.000]. Thus, experiencing any of these events did not predict changes in life satisfaction across years compared to the changes in life satisfaction observed in the matched control group who did not report the event.

**TABLE 3:** Descriptive means and standard errors of well-being outcomes across conditions for traumatic interpersonal events, job loss, and birth.

	Traumatic interpersonal events				Job loss				Birth			
	Event group		Control group		Event group		Control group		Event group		Control group	
Outcome	2018	2019	2018	2019	2018	2019	2018	2019	2018	2019	2018	2019
Life satisfaction	4.81 (0.04)	4.87 (0.04)	5.07 (0.04)	5.15 (0.04)	4.97 (0.04)	5.04 (0.04)	5.23 (0.04)	5.24 (0.04)	5.58 (0.03)	5.53 (0.03)	5.43 (0.03)	5.37 (0.03)
Felt belongingness	4.69 (0.04)	4.73 (0.04)	4.99 (0.04)	4.99 (0.04)	4.96 (0.03)	4.92 (0.03)	5.10 (0.03)	5.09 (0.03)	5.34 (0.03)	5.25 (0.03)	5.25 (0.03)	5.21 (0.03)
Self-esteem	4.70 (0.04)	4.73 (0.04)	4.86 (0.04)	4.86 (0.04)	4.89 (0.04)	4.94 (0.04)	5.04 (0.04)	5.04 (0.04)	5.28 (0.04)	5.25 (0.04)	5.17 (0.04)	5.13 (0.04)
Meaning in life	5.24 (0.04)	5.24 (0.04)	5.27 (0.04)	5.29 (0.04)	5.28 (0.03)	5.31 (0.03)	5.44 (0.03)	5.45 (0.03)	5.73 (0.03)	5.67 (0.03)	5.48 (0.03)	5.45 (0.03)
Gratitude	5.77 (0.03)	5.78 (0.03)	5.88 (0.03)	5.89 (0.03)	5.81 (0.03)	5.87 (0.02)	5.95 (0.03)	5.95 (0.02)	6.05 (0.02)	6.05 (0.02)	6.01 (0.02)	6.02 (0.02)

Descriptives are based on estimated marginal means.

### Felt belongingness

The same analytic strategy was applied to test whether experiencing an event or not interacted with the year assessed (2018 vs. 2019) to predict felt belongingness for each of three event categories (see Figure 1 and Table 3). Significant main effects of event occurred for traumatic interpersonal events [*F*_(1,2058)_ = 35.13, *p* < 0.001, η_p_^2^ = 0.017] and job loss [*F*_(1,2720)_ = 14.79, *p* < 0.001, η_p_^2^ = 0.005], but not for birth [*F*_(1,2448)_ = 2.67, *p* = 0.103, η_p_^2^ = 0.001], with the event groups having lower felt belongingness. than the control group. The main effect for year was not significant in the model examining traumatic interpersonal events [*F*_(1,2058)_ = 0.92, *p* = 0.337, η_p_^2^ = 0.000] or job loss [*F*_(1,2720)_ = 3.15, *p* = 0.076, η_p_^2^ = 0.001], but was significant for the model examining birth [*F*_(1,2448)_ = 17.97, *p* < 0.001, η_p_^2^ = 0.007]. In the birth model, participants had lower felt belongingness in 2019 compared to 2018. However, similar to life satisfaction, the event × year two-way interaction was not significant for all three event categories: traumatic interpersonal events [*F*_(1,2058)_ = 0.91, *p* = 0.341, η_p_^2^ = 0.000], job loss [*F*_(1,2720)_ = 0.75, *p* = 0.388, η_p_^2^ = 0.000], and birth [*F*_(1,2448)_ = 2.14, *p* = 0.144, η_p_^2^ = 0.001]. These results indicate that the difference (or lack thereof) between 2018 and 2019 was similar for people experiencing any of these three types of events and demographically matched controls who did not experience the event.

### Self-esteem

We also investigated whether experiencing an event or not and the year of assessment interacted to predict self-esteem for each event type (see Figure 1 and Table 3). The main effects of event were not significant for traumatic interpersonal events [*F*_(1,2058)_ = 5.99, *p* = 0.014, η_p_^2^ = 0.003], job loss [*F*_(1,2720)_ = 6.26, *p* = 0.012, η_p_^2^ = 0.002], and birth [*F*_(1,2448)_ = 6.39, *p* = 0.012, η_p_^2^ = 0.003]. The main effect of year was also not significant for the models examining traumatic interpersonal events [*F*_(1,2058)_ = 0.83, *p* = 0.362, η_p_^2^ = 0.000], job loss [*F*_(1,2720)_ = 2.02, *p* = 0.155, η_p_^2^ = 0.001], and birth [*F*_(1,2448)_ = 3.26, *p* = 0.071, η_p_^2^ = 0.001]. Critically, the event × year two-way interaction was not significant for all event types: traumatic interpersonal events [*F*_(1,2058)_ = 0.68, *p* = 0.410, η_p_^2^ = 0.000], job loss [*F*_(1,2720)_ = 1.58, *p* = 0.208, η_p_^2^ = 0.001], and birth [*F*_(1,2448)_ = 0.06, *p* = 0.809, η_p_^2^ = 0.000]. These results indicate that differences (or lack thereof) in levels of self-esteem between years did not differ depending on if people experienced an event or not for all three event types.

### Meaning in life

Next, we examined whether experiencing an event or not interacted with the year assessed (2018 vs. 2019) to predict meaning in life for each event category (see Figure 1 and Table 3). Significant main effects of event were found for job loss [*F*_(1,2720)_ = 12.58, *p* < 0.001, η_p_^2^ = 0.005] and birth [*F*_(1,2448)_ = 32.89, *p* < 0.001, η_p_^2^ = 0.013], except for traumatic interpersonal events which was not significant [*F*_(1,2058)_ = 0.57, *p* = 0.449, η_p_^2^ = 0.000]. The event group for job loss had lower meaning in life than the control group, whereas the birth event group was higher in meaning in life than the control group. The main effect of year was also significant for the model examining birth [*F*_(1,2448)_ = 7.16, *p* = 0.008, η_p_^2^ = 0.003], but not significant for the models examining traumatic interpersonal events [*F*_(1,2058)_ = 0.23, *p* = 0.632, η_p_^2^ = 0.000] and job loss [*F*_(1,2720)_ = 0.76, *p* = 0.383, η_p_^2^ = 0.000]. For the birth model, meaning in life declined from 2018 to 2019. However, the critical event × year two-way interactions were not significant for all three event types: traumatic interpersonal events [*F*_(1,2058)_ = 0.32, *p* = 0.573, η_p_^2^ = 0.000], job loss [*F*_(1,2720)_ = 0.18, *p* = 0.673, η_p_^2^ = 0.000], and birth [*F*_(1,2448)_ = 0.45, *p* = 0.504, η_p_^2^ = 0.000]. These results indicate that, relative to demographically matched controls, experiencing an event did not predict changes in people’s level of meaning in life across years.

### Gratitude

Lastly, we investigated if experiencing (versus not experiencing) an event interacted with year (2018 vs. 2019) to predict gratitude across three event categories (see Figure 1 and Table 3). The main effects of event on gratitude were significant for traumatic interpersonal events [*F*_(1,2058)_ = 9.42, *p* = 0.002, η_p_^2^ = 0.005] and job loss [*F*_(1,2720)_ = 12.12, *p* < 0.001, η_p_^2^ = 0.004], except for birth which was not significant [*F*_(1,2448)_ = 1.54, *p* = 0.215, η_p_^2^ = 0.001], with the event groups having lower levels of gratitude than the matched controls. There were no significant main effects of year for the models examining traumatic interpersonal events [*F*_(1,2058)_ = 0.48, *p* = 0.487, η_p_^2^ = 0.000], job loss [*F*_(1,2720)_ = 5.21, *p* = 0.022, η_p_^2^ = 0.002], and birth [*F*_(1,2448)_ = 0.05, *p* = 0.817, η_p_^2^ = 0.000]. However, the event × year two-way interaction was not significant for all three categories: traumatic interpersonal events [*F*_(1,2058)_ = 0.02, *p* = 0.904, η_p_^2^ = 0.000], job loss [*F*_(1,2720)_ = 4.59, *p* = 0.032, η_p_^2^ = 0.002], and birth [*F*_(1_,_2448)_ = 0.06, *p* = 0.802, η_p_^2^ = 0.000]. These results indicate that differences (or lack thereof) in levels of gratitude between years did not differ depending on if people experienced an event or not for all three event types.

## Discussion

Using longitudinal panel data assessing annual change in well-being from 2018 to 2019, the current study compared matched samples of people who experienced a major life stressor relative to propensity score matched controls who did not experience the event across three distinct categories: traumatic interpersonal events, job loss, and birth, and five well-being outcomes: life satisfaction, felt belongingness, self-esteem, meaning in life, and gratitude. In doing so, the current study addressed gaps in existing longitudinal research that limit conclusions about resiliency or growth following stressful events, including the restricted range of outcome measures when examining resilience (Infurna and Jayawickreme, 2019), the focus on one specific adverse event or cumulative adversity scores hindering comparisons between different types of events (Rakhshani and Furr, 2021), and the lack of matched controls to establish the causal role of life events (Mangelsdorf et al., 2019). Our findings showed that for all three classes of events, there were no consistent differences in the five well-being measures (life satisfaction, felt belongingness, self-esteem, meaning in life, and gratitude) between 2018 and 2019 for people who experienced traumatic interpersonal events, job loss, or birth compared to demographically matched controls. These findings indicate high population levels of psychological resilience across several well-being domains in the year following various life events.

Our findings align with prior research that showed stability in a variety of outcomes following an event (Milojev et al., 2014; Chopik et al., 2021, 2022; Blackie and McLean, 2022; Fassbender et al., 2022; Forgeard et al., 2022; Jayawickreme et al., 2022; Reitz et al., 2022) and thus provide further evidence that stability in well-being is the more typical response to major life events (e.g., Infurna et al., 2022; Laceulle et al., 2022; Serrano et al., 2022). Furthermore, in line with growing longitudinal evidence, our results fail to detect evidence for possible post-traumatic growth following major life stressors, insofar as such growth might relate to a broad range of different aspects of well-being (Davis et al., 2021; Rakhshani and Furr, 2021; Dorfman et al., 2022; Infurna et al., 2022). Our findings also align with the few studies that implement matched controls and found notable similarities between those who did and did not experience the event (e.g., Costanzo et al., 2009; van Scheppingen et al., 2016; van Scheppingen and Leopold, 2020). Overall, our results suggest that well-being was relatively unaffected over time by experiencing an event relative to matched controls across three event types and five well-being indicators.

### Implications

Our study extends prior research to provide firmer conclusions regarding the impact of life events on well-being by overcoming methodological limitations present in recent research assessing longitudinal change following adversity from a growth or resilience perspective. First, our study extends prior research that assesses resilience using one outcome (see Infurna and Jayawickreme, 2019) by examining changes in well-being across five constructs that are sensitive to change and represent the broad array of well-being measures used in prior research: life satisfaction, felt belongingness, self-esteem, meaning in life, and gratitude. Overall, our results suggest that all five well-being indicators remained relatively stable over the year assessed for all three event categories, indicating high levels of psychological resilience 1 year on following a stressor. Therefore, our results would suggest that resilience, when it occurs, may be wide-ranging across well-being domains rather than specific to one domain (e.g., life satisfaction). However, our findings contrast those of Infurna and Luthar (2017) who found higher resilience in life satisfaction compared to affective well-being and health outcomes. The different outcomes used in our study may explain the contrasting findings and highlight the importance of using a variety of different outcomes (including those beyond well-being, see Weststrate et al., 2022) in future research to assess resilience across various psychological domains following an event (Infurna and Jayawickreme, 2019). Furthermore, our findings do not suggest that every person will be resilient following a stressor, and it may be that the people who were most negatively impacted by an event dropped out of the survey and were not included in our analyses (Reitz et al., 2022). We encourage future research to replicate our findings using different samples to understand how wide-spread resilience and growth (or lack thereof) is depending on the idiosyncrasies of different people and contexts.

Our study also extends prior literature that has primarily focused on one singular adverse event or cumulative adversity scores (Davis et al., 2021; Rakhshani and Furr, 2021) by simultaneously assessing change in well-being following three distinct event categories of traumatic interpersonal events, job loss, and birth. As these three event types vary in domain, valence, and normality (e.g., work vs. family, trauma vs. negative stressor, positive vs. negative), our research was able to integrate literature examining the impact of adversity with those exploring positive experiences to more systematically test whether adversity is required for growth or resilience to occur (Roepke, 2013; Mangelsdorf et al., 2019). Our analyses challenge the oft-made implications that growth is only possible following adverse experiences by revealing high populations levels of resilience across three distinct classes of events with differing characteristics. Therefore, our results suggest that valence, as well as domain and normality, is unlikely to be an important component in growth or resilience following a stressor. Our findings also extend preliminary evidence regarding character strengths (e.g., wisdom) that the level of adversity or event type does not influence resiliency or personal growth insofar as such responses also relate to well-being (for similar results, see Dorfman et al., 2022; Fassbender et al., 2022). Future research should examine if other aspects of an event, such as how it is processed, influences the possibility of growth or resilience following stressful events across various outcomes (Mangelsdorf and Eid, 2015).

Our study also overcomes a critical methodological limitation in prior research assessing growth and resilience following adversity by comparing matched samples of people who experienced the event relative to propensity score matched controls who did not experience the same event. Our research extends a few studies from the broader literature that have recently began implementing matched controls but that primarily focus on relationship transitions and do not account for the presence of other stressors alongside the focal event (Mangelsdorf et al., 2019; e.g., van Scheppingen and Leopold, 2020). Specifically, our study provided a more precise test of the causal role of life events in well-being by matching participants on demographics, personality, and the presence of other stressors (including the other two events of interest), and conducting comparisons across multiple events and outcomes. Our findings reveal that there were no consistent differences in well-being across years between those who did experience the event and matched controls. Therefore, our analyses challenge the presumed casual role of life events in well-being and suggest that experiencing a traumatic interpersonal event, job loss, or birth does not trigger change in well-being over time. Moreover, our findings also indicate that any observed changes in prior studies that lack a control group was more likely due to factors other than the event or normal maturation processes (van Scheppingen and Leopold, 2020). Future research on growth and resilience following major life events should include matched controls to further disentangle which changes are due to the event and which are due to other factors.

### Caveats and future directions

Although our use of longitudinal panel data assessing annual change in well-being from 2018 to 2019 extends prior research that primarily uses cross-sectional study designs to investigate growth (Jayawickreme et al., 2021), sample size and the small number of participants who completed all relevant variables across three timepoints and reported an event precluded the inclusion of a pre-event baseline. However, the main purpose of a baseline measurement is to show that any change was due to the event (Infurna and Jayawickreme, 2019). Our inclusion of a matched control group who did not report experiencing the same event within the specified timeframe effectively allowed us to disentangle the causal role of experiencing a life event and mitigate the potential limitations of not including baseline data (Forgeard et al., 2022). However, if possible, future research should include both pre-event data as well as matched controls to ensure similarities between the event and control group before the event occurred (Forgeard et al., 2022).

Our assessment of longitudinal change also meant that we were able to examine both immediate and longer-term impacts of three classes of stressors on five well-being outcomes. However, our analyses were constrained to assessing annual differences due to the yearly assessment intervals employed by the NZAVS. Moreover, the BISLE asks participants to report any events that occurred in the past year, meaning that we were also unable assess the specific time when the event occurred (see Howard et al., 2022). Consequently, we were unable to investigate more nuanced changes that may occur over shorter time-intervals, as well as make inferences about the exact time since the event in which any changes occurred. It is possible that the high population levels of psychological resilience found in the current study were because our participants had already adapted to the event between the two annual assessment points, potentially due to having a longer time to recover since the event, with any change perhaps being more transient than enduring. Yet, our findings align with Infurna et al. (2022) who used monthly intervals and found stability or declines in well-being following an event. More intensive daily diary studies are needed to capture nuanced within-person changes as they happen (Blackie et al., 2017; Jayawickreme et al., 2022). For example, future studies could employ experience sampling methods to capture daily fluctuations in functioning or adjustment that do not rely on recall and memory (Blackie et al., 2017; Jayawickreme et al., 2022). A wider range of studies assessing longitudinal change at different time intervals and with specific reference to when the event occurred will allow for deeper understanding of the timeframe in which any change (or lack thereof) is classified as growth or resilience and if the timeframe differs across people (Infurna and Jayawickreme, 2019).

To understand how different types of events impact well-being, we focused on each of the event categories in isolation and did not assess the effect of multiple events in one category or combinations of event occurrence across the three categories (e.g., job loss and birth together). It may be that when these event categories are considered individually, they do not have much effect on well-being. Furthermore, life events often do not occur in isolation and people often experience several events that may differ in domain, severity and/or valence during the same timeframe (Carr and Umberson, 2013). In our current study, about 200 participants (5.53%) were repeated in our analyses of each event type as they reported experiencing an event in more than one of the three event categories we examined. It is possible that experiencing multiple events (either in the same or different domains) may create more capacity for growth or resilience to occur than when only one event type is considered (Jirek and Saunders, 2018; Rakhshani and Furr, 2021). Therefore, future research should assess how different combinations of events (e.g., a positive and negative event) work together in increasing or decreasing the likelihood of growth or resilience.

Due to limited space available in the NZAVS, the BISLE only assesses the reported occurrence of a wide range of life events from the past year (see Howard et al., 2022). Therefore, we based our characterization of the three distinct event categories examined in the current study on how they are typically categorized in the literature (e.g., birth as a positive life event, trauma as defined in the DSM-5). However, this meant that we did not assess people’s subjective experiences of the events, which may differ from our categorization (see Luhmann et al., 2021; Rakhshani et al., 2022, for more information on perceptions of life events). For example, some people may have had traumatic or negative birth experiences and categorize it as such. Furthermore, Boals (2010) found that people’s subjective ratings of the personal impact of an event were more predictive of growth than whether the event was clinically categorized as a traumatic event. This variability may also occur across the specific events included in each category we examined (e.g., job resignation as a more positive event compared to involuntary job loss). Although some within-category variability is expected because the BISLE categories are used as *a priori* based on general clusters of common life events (see Howard et al., 2022), examining event categories was a strength in our approach as we were able to capture a variety of events using a simple indicator to assess changes in well-being following different types of life events. Nonetheless, future research should extend our study by examining annual differences in well-being following events that are subjectively measured by people themselves.

Restricted space in the NZAVS also meant that the well-being outcome measures used in our study are necessarily based on short-form scales (i.e., two or three items). However, this meant that the internal reliability for some of our short-form scales were reasonably low (felt belongingness–T10 and T11: α = 0.60, and gratitude–T10: α = 0.53, T11: α = 0.55). However, to ensure that the averages from both versions of the scales are highly similar, we conducted further analyses using scale validation data collected by NZAVS researchers (for more information, see Sibley, 2022). This dataset includes data from approximately 6,000 undergraduate students across New Zealand universities, in which the foundation sample (*n* = 1,821) completed a random selection of roughly 66% of the items from the full version of each scale covered in the NZAVS. Our analyses revealed strong correlations between the full and short-form versions of the scales for felt belongingness (*n* = 5,574; *r* = 0.79, *p* < 0.001) and gratitude (*n* = 5,566; *r* = 0.87, *p* < 0.001). Therefore, although these lower levels of internal reliability may have attenuated our effect sizes, these measures still appropriately map onto the examined constructs.

## Conclusion

The current study employed longitudinal panel data comparing annual change from 2018 to 2019 for matched samples of people who experienced a major life stressor relative to propensity score matched controls who did not experience the same stressor. We compared matched samples across three distinct events categories: traumatic interpersonal events, job loss, and birth, and five self-report well-being outcomes: life satisfaction, felt belongingness, self-esteem, meaning in life, and gratitude. Results revealed that all five well-being indicators remained consistent across the two assessment points for all three classes of events, with no significant differences over time between those who experienced the event and demographically matched controls. These findings indicate high population levels of psychological resilience in the year following various life events. Analyses also failed to detect significant evidence for possible post-traumatic growth following such events, insofar as such growth might relate to a range of different aspects of well-being. In sum, our findings indicate that most people display high levels of resilience (at least within the timeframe of a year) following different types of life events.

## Data availability statement

The datasets presented in this article are not readily available because ethical restrictions and the need to protect the confidentiality of study participants prevent public deposition of raw data. The data described in this manuscript are part of the New Zealand Attitudes and Values Study (NZAVS). Full copies of the NZAVS data files are held by all members of the NZAVS management team and advisory board. A de-identified dataset containing the variables analyzed in this manuscript is available upon request from the corresponding author, or any member of the NZAVS advisory board for the purposes of replication or checking of any published study using NZAVS data. The full statistical standard for life events in the NZAVS (with coding details and generalized examples of responses) is provided under the supplementary information for the inventory article (Howard et al., 2022) available at https://osf.io/75snb/. The Mplus syntax used to test all models reported in this manuscript are available on the NZAVS website: www.nzavs.auckland.ac.nz (see also: https://osf.io/75snb/). Requests to access the datasets should be directed to CGS, c.sibley@auckland.ac.nz.

## Ethics statement

The studies involving human participants were reviewed and approved by The University of Auckland Human Participants Ethics Committee (Reference Number: 014889). The patients/participants provided their written informed consent to participate in this study.

## Author contributions

CH conceptualized the study, performed the statistical analysis, wrote the manuscript, and performed the manuscript revision. All authors contributed to the design of the study. CGS organized the database and funding for the study. CGS and NCO provided supervision and extensive feedback on the manuscript.
